# Farm Vehicle Following Distance Estimation Using Deep Learning and Monocular Camera Images

**DOI:** 10.3390/s22072736

**Published:** 2022-04-02

**Authors:** Saeed Arabi, Anuj Sharma, Michelle Reyes, Cara Hamann, Corinne Peek-Asa

**Affiliations:** 1 Department of Civil, Construction, and Environmental Engineering, Iowa State University, Ames, IA 50011, USA; anujs@iastate.edu; 2 National Advanced Driving Simulator, University of Iowa, 127 NADS, Iowa City, IA 52242, USA; michelle-reyes@uiowa.edu; 3 Department of Epidemiology, University of Iowa, 145 N Riverside Dr, S449 CPHB, Iowa City, IA 52242, USA; cara-hamann@uiowa.edu; 4 Injury Prevention Research Center, University of Iowa, 145 N Riverside Dr, Iowa City, IA 52242, USA; corinne-peek-asa@uiowa.edu; 5 Department of Occupational and Environmental Health, University of Iowa, 145 N Riverside Dr, S143 CPHB, Iowa City, IA 52241, USA; 6 Office of Research Affairs, University of California San Diego, 9500 Gilman Drive, #0043, La Jolla, CA 92093, USA

**Keywords:** safety, driving behavior, distance estimation, deep learning

## Abstract

This paper presents a comprehensive solution for distance estimation of the following vehicle solely based on visual data from a low-resolution monocular camera. To this end, a pair of vehicles were instrumented with real-time kinematic (RTK) GPS, and the lead vehicle was equipped with custom devices that recorded video of the following vehicle. Forty trials were recorded with a sedan as the following vehicle, and then the procedure was repeated with a pickup truck in the following position. Vehicle detection was then conducted by employing a deep-learning-based framework on the video footage. Finally, the outputs of the detection were used for following distance estimation. In this study, three main methods for distance estimation were considered and compared: linear regression model, pinhole model, and artificial neural network (ANN). RTK GPS was used as the ground truth for distance estimation. The output of this study can contribute to the methodological base for further understanding of driver following behavior with a long-term goal of reducing rear-end collisions.

## 1. Introduction

Road traffic injuries are among the eight main causes of death, according to the World Health Organization [1]. Rear-end collisions are one of the most frequent among the various types of crashes and account for 6.7 percent of fatalities and injuries yearly [2]. Several factors contribute to the occurrence of rear-end crashes, such as vehicle types, road conditions, and driver characteristics. There are numerous studies that have applied deep learning methods to analyze the underlying factors that may contribute to crashes [3,4,5,6,7,8,9,10].

The National Motor Vehicle Crash Causation Survey (NMVCCS) found possible driver contribution for 94% of crashes [11]. The most common driver attributed factors were recognition errors, including driver inattention and distraction.

To mitigate the risk of rear-end collisions, driver assistance systems that can reliably predict the collision and provide timely warnings have been developed [12]. The systems estimate the relative distance to the vehicle ahead. Then, time-to-collision (TTC) is calculated based on the estimated relative distance and the vehicles’ speeds, and if the calculated TTC is less than a certain threshold, the collision warning will be issued [13].

To calculate the TTC, the relative distance to the vehicle ahead should be estimated as accurately as possible. Several methods utilizing various types of sensors have been introduced for distance estimation. For example, radar sensors, which are commonly used to estimate depth ranges, are especially beneficial in adverse weather conditions and poor illumination conditions [14,15]; however, these sensors are relatively expensive. Vision-based FCW systems have been investigated as a lower-cost alternative to radar, in which they use cameras to detect the vehicle ahead and provide the necessary warnings to the driver to avoid rear-end crashes [16,17,18,19]. Unlike radar sensor data, image data do not contain depth information. The depth of the objects captured in the image can be estimated by relating the size of the objects present in the image to their size in the real world, as the height of an object in the image is inversely proportional to its distance from the camera [20,21,22,23,24].

Several methods have been used to extract object depth information from image data. Generally, there are two main vision-based methods for depth estimation: stereo- and monocular-vision approaches. The former uses multi-view geometry and stereo image pairs to rebuild a 3D space and generate the depth information of the target. However, errors and computational complexities from the calibration and matching of stereo image pairs reduce the measurement accuracy and efficiency. Monocular-vision methods, however, have certain advantages, such as being less expensive, having a simple hardware structure, and a wide field of application.

Generally, monocular-vision methods for distance estimation can be divided into two categories. In the first category, the distance estimation is conducted based on the geometric relationship and camera-imaging model [25]. In these types of methods, several parameters from the camera (e.g., the elevation of the camera and the measured object; the height of the target vehicle) need to be provided in advance. Liu et al. used the geometric positional relationship of a vehicle in the camera coordinate system to construct the correspondence between the key points in the world coordinate system and the image coordinate system and then they established a ranging model to estimate the target vehicle distance [25]. Kim et al. used the camera imaging model and the width of the target vehicle to estimate the distance to a moving vehicle that is far ahead [22].

The second category involves constructing a regression model using machine learning. Wongsaree et al. trained a regression model using the correspondence between different positions in an image and their corresponding distances to complete distance estimation [26]. Gökçe et al. used the target vehicle information to train a distance regression model for distance estimation [27]. The main disadvantage of these methods is that they have to collect a large number of training data with real distances.

The primary objective of this study is to develop and validate a distance estimation method using monocular video images recorded by a custom data collection device that was designed to study driver behavior while approaching, following, and overtaking farm equipment traveling in the same direction. Two factors make this applied situation novel. First, existing vehicle TTC estimates are based on calculations from a forward-facing system assessing an object that the equipped vehicle is approaching. The unique nature of our question required the opposite—a rear-facing system estimating distance from an object approaching the equipped vehicle. Second, farm equipment, which has a wide range of size and operational features, behaves differently in roadway interactions than passenger vehicles, potentially influencing which estimates are most valid. The devices are mounted on several farm vehicles to investigate driver-following behavior to collect data over many seasons. To better manage the data, the captured videos are compressed and have low resolution. Consequently, some methods, such as distance estimation based on the license plate [28], cannot be applied. Moreover, frequent calibration of the camera and monitoring of calibration quality is not practical since the device is mounted on the vehicles which routinely travel over rough terrain. As a result, stereo-based distance estimation is not practical. Additionally, since there will be a large number of vehicles instrumented with these devices, the cost per unit should be reasonable, eliminating the option of using more expensive sensors such as LiDAR and/or radar.

Therefore, to confirm the distance estimation method, an experiment was designed in which two pairs of vehicles were instrumented with the study devices and RTK GPS sensors. Several trials of vehicle interactions were conducted on a closed course, and GPS data and video footage were captured. The data were then aggregated, cleaned, and processed by employing the Nvidia DeepStream object detection framework [29]. Using the output of detection, three different distance estimation models, i.e., linear regression, pinhole, and artificial neural network (ANN), were applied and their results were compared. The accuracy of the proposed methods was verified by comparing with RTK GPS-based estimated distances, which have sub-inch accuracy.

## 2. Methodology

### 2.1. Data Collection Device

**Figure 1:**
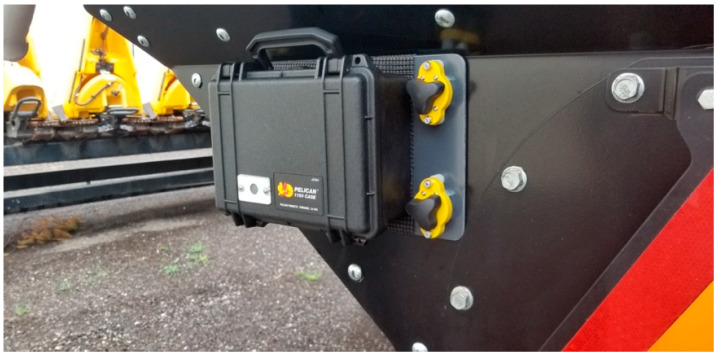
Data collection device mounted on the back of a piece of farm equipment.

The data collection devices were designed specifically for a naturalistic study of how drivers approach, follow, and pass farm equipment on the roadway. Contained in rugged, weather-resistant cases approximately 0.23 m × 0.20 m × 0.10 m, the devices attach to farm equipment using switch magnets. Video data were recorded at a frequency of 30 Hz and a resolution of 800 × 600 pixels. Figure 1 depicts the data collection device.

### 2.2. Validation Data Collection

Validation data were collected on a closed runway about 1000 feet long and 150 feet wide. A lead vehicle was equipped with several devices in a vertical stack such that the camera lenses were at different heights (0.71 m to 2.02 m) to approximate the range of heights from common farm vehicles (e.g., combines, tractors). The devices were set to record continuously. A Trimble R8 RTK GPS receiver was mounted directly above the stack of devices. The Trimble R8 is rated with a horizontal accuracy of ±0.03 feet (8 mm). Real-time corrections were provided via cellular modem by the Iowa Real-Time Network (IaRTN), a statewide system of base stations operated by the Iowa Department of Transportation (IDOT). In the experience of the research center that provided the RTK equipment, the horizontal accuracy of the IaRTN corrections in practice is approximately ±0.05 feet (15 mm). The RTK was recorded at 1 Hz.

**Figure 2:**
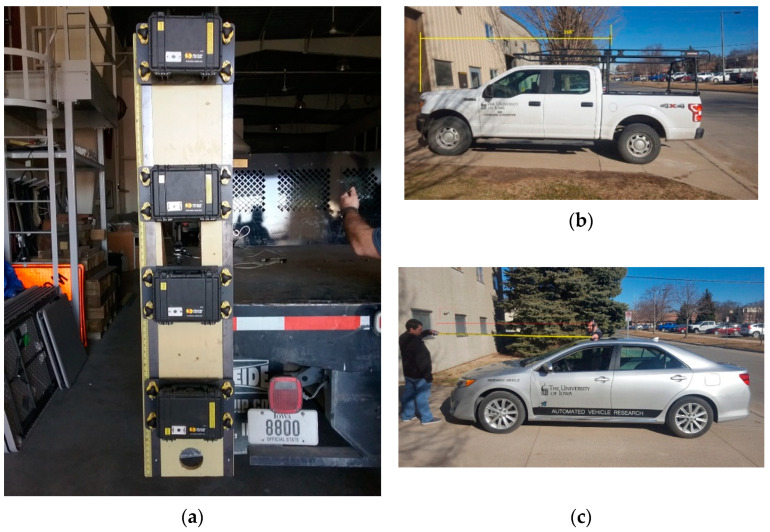
Instrumented vehicles: (**a**) data collection devices mounted vertically at four different elevations on the back of the lead vehicle, (**b**) following truck vehicle, and (**c**) following sedan vehicle.

Another identical Trimble R8 receiver was mounted above each following vehicle. Two different types of following vehicles were used in the data collection: a 2012 Toyota Camry sedan and a 2018 Ford F150 SuperCrew pickup truck. For the sedan, the mounting pole was extended through the sunroof of the cab and, relative to a driver’s perspective, was located 4 inches right of center and 92 inches behind the front bumper. For the pickup, the mounting pole was secured to an equipment rack behind the cab, 29 inches right of center and 166 inches behind the front bumper. Figure 2 shows the instrumented vehicles.

Approximately 40 trials were recorded with each of the following vehicles traveling behind the lead vehicle. For each trial, the driver of the lead vehicle would begin to travel down the runway and attempt to quickly accelerate to and then maintain a consistent speed of about 30 mph or 40 mph. The driver of the following vehicle attempted a wide variety of maneuvers, including following at various time headways (i.e., 1, 3, and 5 s), changing time headways while following, changing lanes, and passing.

### 2.3. Distance Estimation Models

Three vision-based distance estimation models were evaluated: linear regression, pinhole, and ANN. Since the image data do not contain the depth information of objects within them, that information should be estimated by using the size and position of the objects in the image. To this end, Nvidia DeepStream [30], a deep-learning-based vehicle detection framework, was used to extract object position and size (i.e., bounding box information) in the image space.

**Figure 3:**
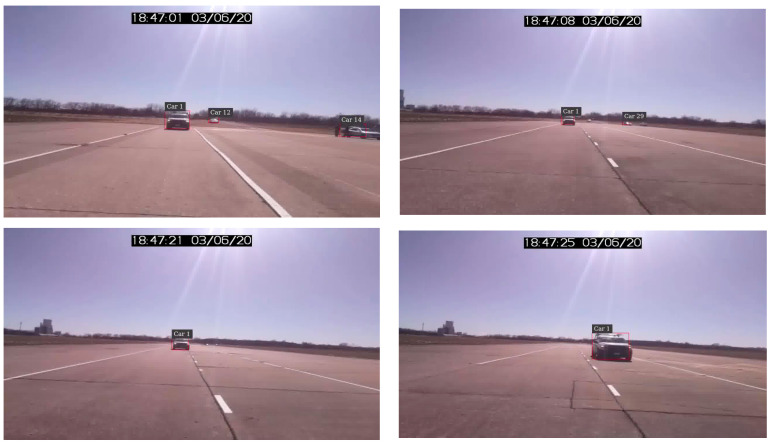
A sample of DeepStream vehicle detection.

DeepStream is a complete streaming analytics toolkit for AI-based video and image understanding, as well as multi-sensor processing. It uses the open-source multimedia handling library GStreamer to deliver high throughput with a low-latency streaming processing framework. The DeepStream SDK is based on the open-source GStreamer [29] multimedia framework. A DeepStream application is a set of modular plugins connected in a graph. Figure 3 shows a sample of DeepStream output from our study dataset. It should be noted that some of the DeepStream output were sampled, and its accuracy was confirmed by the authors to be within an acceptable range. It should be noted that the bounding box size is small when the vehicle is far from the farm equipment. However, the focus of this study was to develop a system to investigate the driving behavior when the vehicles are following and preparing to overtake the farm equipment. For the distances observed in these situations, the bounding box is large enough to reasonably estimate the distance.

The detection outputs were then used to estimate the vehicle distance d. Detection outputs include bounding box height (H), width (W), bounding box center vector $(l_{x}$ and $l_{y}),\;$originated from the upper left of the video frame, and type of the vehicle, i.e., pickup truck or sedan. Detection outputs, along with the height of the camera lens, were used to train the distance estimation models.

#### 2.3.1. Linear Regression

The first distance estimation model is linear regression. Four different models, each devoted to the data collected from one of the data collection devices of differing heights, were fitted using the following equation:

(1) y=Xβ+ε

where y and ε are n×1 vectors of the response variables, i.e., estimated distances, and errors of n observations, and X is an n×p design matrix.

#### 2.3.2. Pinhole Camera Model

The second distance estimation model presented here is a pinhole camera model [25].

**Figure 4:**
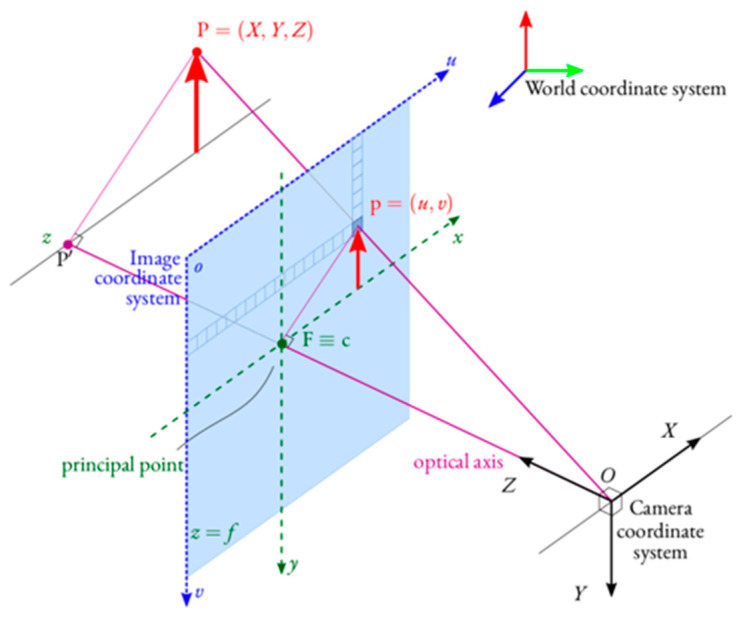
Pinhole camera model.

Let *P* = [*X* *Y* *Z*]*^T^* be an arbitrary 3D point seen by a camera placed at the origin *O* of its camera space OXYZ, and *p* = [*u* *v*]*^T^* be the image of *P*, expressed in the image coordinate system ouv. The point *p* represents a pixel in an image captured by the camera, which is formed by intersecting the light ray from *P* passing through the camera optical center *O* and the image plane. Assuming that the projective plane is perpendicular to the *Z*-axis of the camera coordinate system, the intersection is at the principal point *F* = [0 0 *f*]*^T^*, which is expressed in the image coordinate system as *c* = [*c_x_* *c_y_*]*^T^*. Figure 4 illustrates the pinhole camera model.

The distance of the object P from the center of the camera *O* can be calculated using the following equation:

(2) $$d=\frac{\sqrt{A^{2}+B^{2}+1}}{Z},\;A=\frac{u-c_{x}}{f}\;,\;B=\frac{v-c_{y}}{f}$$

Since, in the current study, the following vehicle was near the center of the camera image, Equation (2) can be simplified to

(3) $$d=f\cdot \frac{h}{H}$$

where f is the focal length of the camera, h is the height of the vehicle in the real world, and H is the height of the vehicle in the image space in pixel values. Moreover, since the focus of the current study is on sedan cars and pickup trucks, these two types of vehicles were considered, and the average of their heights were measured to find h. The actual heights for the sedan and the pickup truck were 57 inches and 76 inches, respectively. The camera focal length, f, was calculated by conducting a calibration process which involved determining the relationship between the height of the detected object in the image space, H, and its distance from the camera in the real world, h, by using the pinhole camera model (Equation (3)). To this end, several peaks and valleys of GPS-based distance estimation along with its corresponding H value, derived from detection processing, were considered. The calibration process included relating the H values to the GPS-based distances using Equation (3). After conducting the calibration, the focal length of 892 pixels was calculated.

#### 2.3.3. Artificial Neural Network (ANN)

Finally, an ANN structure was designed to regress the distances by considering the detection results as the inputs of the network. The network consisted of the *input layer*, which had six neurons (equal to the number of variables used for training), the *output layer*, which had one neuron (estimated distance), and *hidden layers*, which connect the *input layer* to the *output layer*. The number of *hidden layers* and the number of neurons in each of them are two tuning parameters. Moreover, a *dropout* layer was considered for the last *hidden layer*. The idea of *dropout* is to randomly (with a specific rate) drop neurons along with their connections from the neural network to prevent overfitting. In addition, activation functions were used to increase the nonlinearity of the neural network. In this study, we applied two well-known activation functions, i.e., *relu* and *tanh*. *Batch size* is the number of data points on which the training will be conducted. Finally, the *epochs* are the number of times that the training will be conducted.

Since there is no exact solution to find the optimal network architecture and configuration, an exhaustive grid search was conducted to find the best network based on the regression accuracy metric. The grid search was conducted between first hidden layer nodes ∈{16,8,4}, number of hidden layers ∈{2,3,4,5}, last hidden layer nodes ∈{4,5}, dropout rate ∈{0.1, 0.2, 0.3, 0.4, 0.5, 0.6, 0.7,0.8,0.9}, activation functions ∈{relu, tanh}, batch size∈{8,16,32}, and epochs ∈{100,300,800}.

#### 2.3.4. Comparison of GPS-Based and Video-Based Distance Estimation

**Figure 5:**
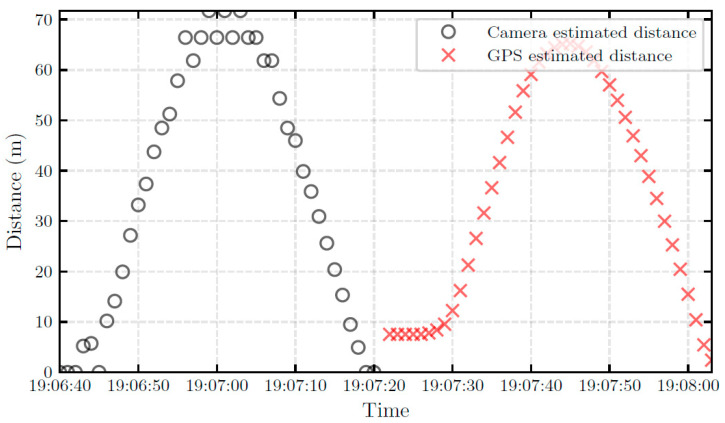
Comparison of the GPS and camera distance estimation.

To investigate the accuracy of the distance estimation models, the ground truth distances, i.e., GPS-based distances, were compared with distances derived using the detection results from the pinhole model. Figure 5 depicts the GPS-estimated and pinhole model-estimated distances. The video-based distance time series and GPS-based time series were recorded at different data frequencies (1 Hz and 30 Hz, respectively). Consequently, in order to quantify the distance estimation error between the two time series, the fast dynamic time warping (FDTW) method was used to find the optimal alignment between them [31]. The FDTW is an approximation of dynamic time warping (DTW) and it has linear time and space complexity.

**Figure 6:**
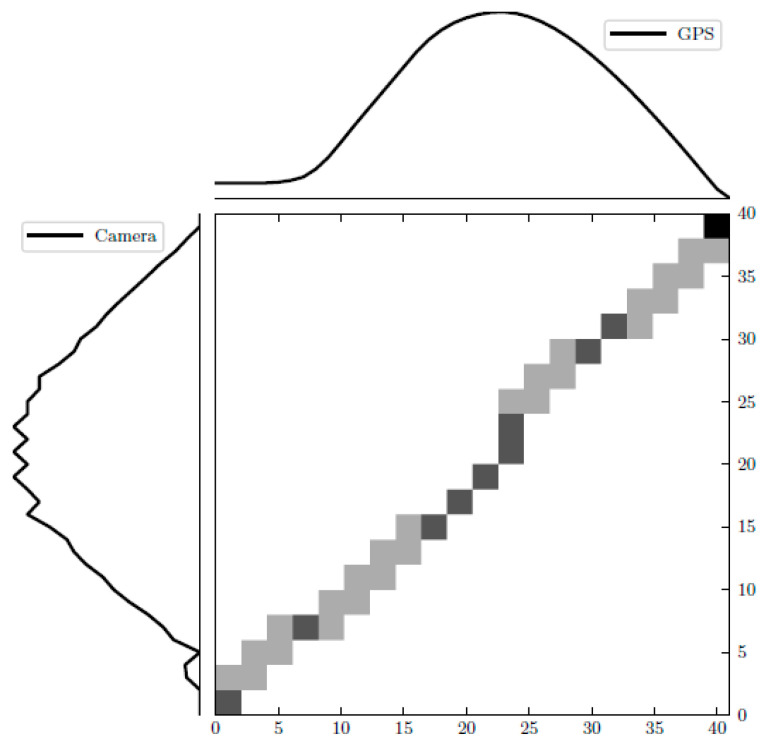
FDTW cost matrix of GPS-based and camera-based estimated distances time series.

A two-dimensional cost matrix *D* was constructed where *D(i*, *j)* was the minimum distance warp path that can be constructed using the two time series. Figure 6 illustrates the cost matrix of the two time series. Using the minimum distance path derived by FDTW, the two time series were aligned.

**Figure 7:**
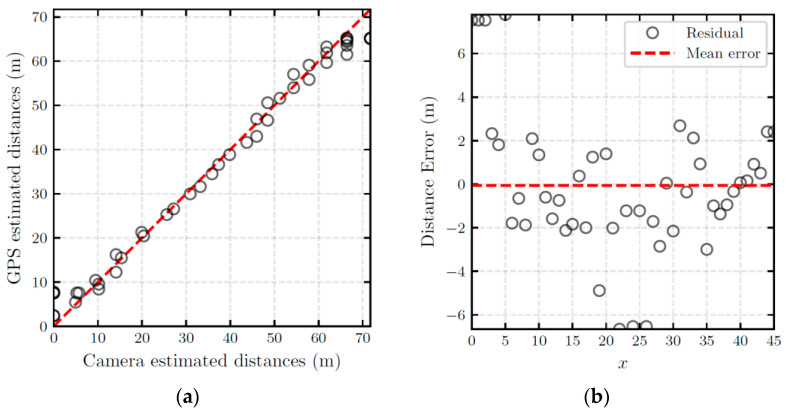
Quantification of pinhole camera-based distance estimation error: (**a**) scatter plot of GPS-and camera-estimated distances; (**b**) plot of the estimation residuals.

Figure 7a shows the scatter plot of GPS and camera estimated distances. It can be seen that the camera distance estimation could successfully estimate the distances obtained by GPS. To quantify the distance estimation error, the residuals were calculated. Figure 7b shows the pattern of residuals. The residuals are roughly randomly scattered, meaning that they are unbiased and homoscedastic. The extreme errors at the middle of the graph show that the camera estimation is less reliable in estimating distances beyond 60 m.

## 3. Results

Once the time series were aligned by using the FDTW, GPS-based distance estimations were used as the “gold standard” labels for training the regression and ANN model. As described in Section 2.3.3, an exhaustive search was conducted to find the most optimal ANN configuration and architecture in the grid search. The review of model accuracy showed that a model with four hidden layers (with layers having 8, 7, 6, and 5 neurons, respectively), no dropout, *relu* activations, batch size of 16, and epochs number of 800 is the most optimal model in the grid search.

**Figure 8:**
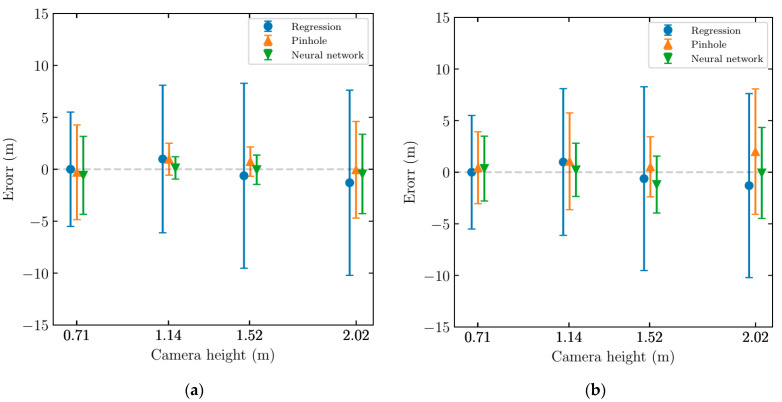
Camera-based distance estimation error for various trials and different units: (**a**) sedan; (**b**) pickup truck.

To further analyze the distance estimation models, the distance errors were calculated for multiple randomly selected trials, considering data collected from the device at each height. Then, the mean and standard deviation of errors were calculated for each device height. Figure 8a,b shows the vision-based distance estimation error with two standard deviations error bars for the sedan and the pickup truck, respectively. The review of results for both the sedan and the pickup truck shows that the distances estimated using the linear regression model have the highest standard deviation while the ANN has the lowest standard deviation overall. The ANN model also has the lowest error, having the closest error to zero. Table 1 summarizes the mean error and standard deviation of error for each distance estimation method for the sedan and the pickup truck.

**Figure 9:**
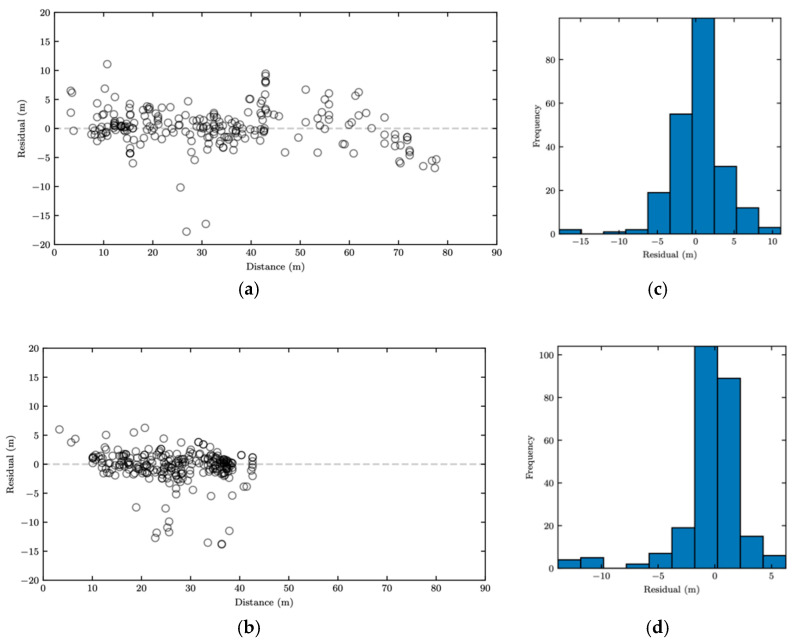
ANN-based following distance estimation: (**a**) residual pattern of estimated distances for the sedan; (**b**) residual pattern of estimated distances for the pickup truck; (**c**) distribution of residuals of estimated distances for the sedan; and (**d**) distribution of residuals of estimated distances for the pickup truck.

Based on the presented results, the model ANN was determined to be the best estimator for distance. Figure 9 shows the scatter plot of residuals, derived from the ANN model plotted against the ground truth, i.e., GPS-based distances, for both the sedan and the pickup truck. As it can be seen, the residuals do not follow any specific pattern overall, indicating that the ANN model provides a good fit to the data. Moreover, the histogram of residuals was investigated. Based on the results shown in Figure 9, the residual histograms for both the sedan and the pickup truck follow the normal distribution with the mean close to zero.

## 4. Discussion

The results of this investigation indicated that using shallow models, for instance, linear regression, were not very effective for distance estimation due to their inconsistency in prediction, i.e., high variance. This seems to be related to the fact that these models cannot identify all the nonlinear relations between the input, i.e., detection outputs, and the output, i.e., estimated distances. Consequently, the artificial neural network model was determined to be the best option for distance estimation with a reasonable standard deviation. Moreover, increased height impacts the standard error far more than the mean, especially for the regression model. This is important because the diversity of farm equipment makes it impossible to normalize camera height, so this analysis will help adjust for height in future models. It should be noted, however, that regardless of the distance estimation method, the results are highly dependent on the quality of the input, i.e., detection outputs. Detection model performance is related to the quality of the video as well as the detection algorithm; thus, to improve the detection results, having high-resolution videos would be helpful. In addition, detection output could be improved by using the transfer learning methods and retraining the model using the actual videos used in this study. In addition to retraining, which could significantly improve the results, a careful data annotation would also make a difference. The review of detection output showed that, sometimes, the entire or some portion of tires were excluded from the bounding box, which might be related to the careless annotation. Finally, since the recording platform will be mounted on farm equipment, it is prone to drastic vibration. The vibration might sometimes cause the captured video to be blurry, and, consequently, the detection algorithm fails. In this case, a vibration resilience enclosure may improve the results.

## 5. Conclusions

In this study, we propose a solution to extract the depth information of objects in an image recorded by a low-resolution monocular camera using the deep-learning-based approach. Video and RTK GPS were collected on a closed course with two types of following vehicles. The data were then aggregated, cleaned, and preprocessed using the Nvidia DeepStream, which is an analytics toolkit for video and image analysis. The vehicle detection and tracking were conducted using DeepStream, and bounding boxes information was obtained. Using the pinhole camera model, the height of detected objects in the image was related to the distance of the object in the real world to the camera. The distance estimation process involved finding the focal length of the camera through a calibration step which was conducted by comparing the distances estimated from pinhole model with that of RTK GPS as the ground truth. In addition to the pinhole model, two more distance estimation models were investigated, i.e., linear regression and artificial neural network (ANN). Among the mentioned three models, the ANN was the best distance estimator by having the least mean error and standard deviation. Finally, the legitimacy of the proposed ANN method was confirmed by investigating the scatter of the residuals and histogram plots. The methodology confirmed in this study can be applied to future study of farm vehicle and passenger vehicle interactions in terms of following distance. The output of this study can be used to inform prevention efforts to reduce the risk of rear-end collisions, especially when a heavy vehicle with large blind spots is involved.

## Figures and Tables

**Table 1:** Comparison of distance estimation error for the three candidate models.

		Mean			Standard Deviation		
	Camera Height (m)	Linear Regression	Pinhole	ANN	Linear Regression	Pinhole	ANN
Sedan	0.71	−0.001	−0.432	0.354	5.507	3.481	3.195
	1.14	0.988	1.055	0.229	7.107	4.689	2.524
	1.53	−0.625	0.522	−1.196	8.905	2.913	2.66
	2.02	−1.298	1.997	−0.071	8.918	6.075	4.50
	**Average mean =**	0.99	1.19	0.17			
Truck	0.71	−0.001	−0.293	−0.740	5.507	4.561	3.693
	1.14	0.988	0.968	0.181	7.107	1.541	1.321
	1.53	−0.625	0.739	−0.368	8.905	1.414	1.349
	2.02	−1.298	−0.048	0.0775	8.918	4.653	3.849
	**Average mean =**	0.49	0.85	0.13			

## Data Availability

Contact the authors to access the data.
